# Frontal Fibrosing Alopecia in Men: A Review of the Literature

**DOI:** 10.3390/jcm14061914

**Published:** 2025-03-12

**Authors:** Ana Melián-Olivera, Adrián Imbernón-Moya, María L. Porriño-Bustamante, Cristina Pindado-Ortega, Daniel Fernandes-Melo, David Saceda-Corralo

**Affiliations:** 1 Dermatology Department, Ramón y Cajal University Hospital, 28034 Madrid, Spain; draanamelian@gmail.com (A.M.-O.); cpindadoortega@gmail.com (C.P.-O.); 2 Hair Disorders Unit, Grupo Pedro Jaén, 28002 Madrid, Spain; dradrianimbernon@gmail.com; 3 Dermatology Department, Severo Ochoa Hospital, 28914 Madrid, Spain; 4 Dermatology Department, La Zarzuela Hospital, 28023 Madrid, Spain; mporrinobustamante@gmail.com; 5 Dermatology Department, State University of Rio de Janeiro, Rio de Janeiro 13414-903, Brazil; danielfernandesmelo@yahoo.com.br

**Keywords:** cicatricial alopecia, frontal fibrosing alopecia, lichen planopilaris, scarring hair loss, male hair loss

## Abstract

**Background:** Frontal fibrosing alopecia (FFA) is a primary cicatricial alopecia, initially described in postmenopausal women but increasingly reported in men. The male form remains under-recognized, often misdiagnosed as androgenetic alopecia (AGA) or alopecia areata (AA), particularly in the beard. **Objective:** This review aims to summarize the current literature on the epidemiology, clinical presentation, etiopathogenesis, diagnosis, and treatment of FFA in men. **Epidemiology and Clinical Features:** FFA in men typically presents at a younger age compared to women. Key features include frontal and temporal hairline recession, early involvement of the beard and sideburns, and a high prevalence of eyebrow alopecia (43–94.9%). Facial papules and body hair loss are more common in men than women. Occipital involvement varies widely across studies (8–45%). Clinical features like beard alopecia, often presenting as plaque or diffuse patterns, are highly suggestive of FFA in men but are not part of current diagnostic criteria. **Etiopathogenesis:** FFA is postulated to have an autoimmune basis influenced by genetic, hormonal, and environmental factors. Genetic studies have identified associations with HLA-B*07:02 and CYP1B1 loci. Environmental triggers include prolonged use of facial sunscreens and moisturizers, as demonstrated in case-control studies and meta-analyses. **Diagnosis:** Diagnosis is predominantly clinical, supported by trichoscopy and biopsy when needed, particularly in cases overlapping with AGA or AA. Unique presentations, such as beard alopecia and the “watch sign”, highlight the importance of considering FFA in atypical male cases. **Treatment:** Current treatment protocols in men mirror those for women and focus on disease stabilization. Oral 5-ARi (dutasteride) combined with topical corticosteroids and calcineurin inhibitors form the first line. Additional treatments include intralesional corticosteroids, oral isotretinoin for facial papules, and minoxidil for associated AGA. Surgical hair transplantation remains controversial, requiring disease control and careful patient counselling. **Conclusions:** FFA in men presents with distinct clinical features and challenges in diagnosis, often overlapping with other alopecia. Further studies are needed to validate diagnostic criteria and evaluate treatment efficacy in this underrepresented population.

## 1. Introduction

Frontal fibrosing alopecia (FFA) is a primary cicatricial alopecia first described by Kossard [1] in postmenopausal women in 1994. It was only in 2002 that the disease was first described in men [2].

To date, cases in the literature do not exceed 500 male patients and are distributed among small case series and cohorts, the longest of which included 55 patients [3]. The percentage of male patients with FFA ranges from 3 to 8.1% [4,5]. However, its incidence is probably underestimated due to misdiagnosis with androgenetic alopecia (AGA) or alopecia areata (AA), especially on the beard [3].

## 2. Epidemiology

The mean age of onset tends to be lower in men [4,6]. In an observational study of 490 patients, 23 of whom were male, Kanti et al. [6] described a mean age at presentation of 49 years in males and 60 years in females, similar to the series of Vañó-Galván et al. [4]. On the other hand, ages at presentation of 17 and 21 years have also been described [7,8,9].

**Figure 1:**
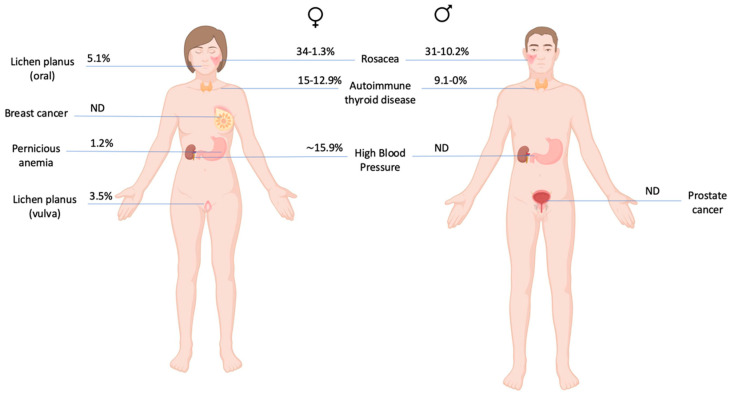
Main comorbidities in frontal fibrosing alopecia in women and men.

There are comorbidities associated to FFA reported in the literature (Figure 1) [10]. In terms of dermatological comorbidities, the coexistence of AGA is more common in males, reaching 83% overlap between entities in some studies [11]. Specifically, in the study conducted by Vañó-Galván [4], a prevalence of 67% was observed, compared to 40% in females. Alternatively, its association with lichen planopilaris (LPP) is variable. Pathoulas et al. reported a series of 55 men with FFA, of which 18 (32%) also had LPP [3]. In the series carried out by Peterson et al. [12], two of the seven patients had associated LPP, and in the study conducted by Kanti et al., the percentage of affected men (25%) was higher than that of women (15%) [6].

*ND*, *Not defined*. Certain differences can be observed between the prevalence of some comorbidities, particularly thyroid diseases. There are no data to suggest an increased incidence of cancer in FFA, but hormonal treatments related to breast cancer have been proposed as possible risk factors for the disease. The situation is similar with prostate cancer, although published case series are even smaller.

Some series, such as that performed by Lobato et al. [13], have described a prevalence of rosacea of up to 31%, similar to the data collected in the female gender (34% in Pindado et al.’s case series) [14]. Another case-control study [9] also found a similar prevalence of rosacea between both genders in their case-control study (12.1 and 10.2%, respectively, in men), but the association was not significant in the males. On the other hand, Kanti et al. [6] showed that the percentage of patients with rosacea was higher in males (13 vs. 1.3%), although the low prevalence in females was striking.

The retrospective study by Trager et al. [5] evaluated the association of comorbidities in subjects with FFA (n = 173 patients, 14 males) without distinguishing between genders. Patients with FFA had a higher risk of hypothyroidism (OR 2.15; 95% CI 1.20–3.87). However, the prevalence of this condition in men varies from 0% [4,8,9,11,15,16,17] to 9.1% [18] in published series, and no association with FFA was found in a case-control study [9]. Data on cardiovascular risk are scarce and mostly relate to patients with LPP and FFA. Ranum et al. [19] observed a reduced cardiovascular risk in 363 patients (43 men) compared with controls, and Trager et al. [5] reported a reduced risk of type 2 diabetes mellitus (306 patients, 70 men), but further studies are needed to clarify this association.

## 3. Etiopathogenesis

The exact pathomechanism of FFA is still unknown, but an autoimmune pathophysiology, probably influenced by hormonal and environmental factors, is postulated to develop in genetically predisposed individuals [20]. A genomic study analyzing the prevalence of the four risk loci identified in females has recently been published analyzing the same loci in 92 male FFA patients [21]. A statistically significant association was found for the loci HLA-B*07:02 (OR 3.01, *p* = 5.4 × 10^−6^), which is involved in antigen presentation, and CYP1B1 (OR 2.36, *p* = 1.2 × 10^−3^), which is related to the metabolism of xenobiotic estrogens.

Several studies have shown cases of familial aggregation [22,23,24,25], mostly in women with sibling relationships, with the prevalence of family history estimated at 5–8% [26]. Regarding familial cases in men, disease presentation between two siblings, a brother and sister, and a father and son has also been described [26,27,28].

On the contrary, the hormonal role in the development of FFA is being studied because of its predominance in females, especially in postmenopausal or early menopausal women [4], and its favorable response to 5-alpha reductase inhibitors (5-ARi) in controlling its progression [29]. To date, 11 patients with prostate adenocarcinoma and FFA have been reported, 4 of whom received hormonal treatment (bicalutamide and goserelin/tryptorelin; estrogens) [9,11,13,15,30,31,32]. In contrast, one patient with panhypopituitarism and replacement therapy developed FFA, although he probably had a strong genetic predisposition as his brother also had the condition [27].

The progressive increase in the incidence of this disease has sparked interest in identifying external factors that trigger its onset.

The case-control study led by Moreno-Arrones et al. [9] analyzed data from 77 men (19 with FFA and 58 controls). The authors demonstrated an association between FFA and the use of facial sunscreens (OR = 11.6; 95% CI 1.7–80.9) and anti-ageing creams (OR = 1.84; 95% CI 1.04–3.23), while the association with the use of moisturizers was not statistically significant (*p* = 0.06). Another study also confirmed a higher exposure to facial moisturizers and sunscreens in 17 men with FFA compared to 73 controls, while no differences were found in the use of hair care products and other facial care products (scrubs, masks, aftershave, cleansers) [33]. In the work of Pathoulas et al. [3] on 270 men with either FFA or LPP, a higher long-term and consistent use of facial sunscreens was found in patients with FFA compared to those with LPP, but there were insufficient data to establish causality.

Another case-control study (451 patients, including 17 men) described associations with the use of facial moisturizers (OR 1.99), facial soaps (OR 2.09) and formalin hair straightening (OR 3.19) [34]. The use of cleansing shampoos (OR 0.35) and smoking (OR 0.33) were suggested as protective factors. No association was found with sunscreens.

A recent meta-analysis analyzed the relative risk from nine studies involving 1248 patients, 72 of whom were male (some of which are part of the studies described above). A statistically significant risk associated with the use of sunscreens (OR 4.61, *p* = 0.006) and facial moisturizers (OR 5.07, *p* = 0.01) in men was found. In women, the OR was lower for sunscreens (OR 2.74, *p* = 0.007) and not significant for facial moisturizers (OR 1.58, *p* = 0.16) [35]. No significant differences were found for other topical facial or hair products.

Several studies have focused on describing the incidence of contact dermatitis in patients with FFA, but the number of men included is small. Pastor et al. [36] studied 36 patients with FFA (35 females and 1 male) who were tested with patch and photo patch tests to a range of allergens and their own cosmetic products. Sensitization to benzyl salicylate was found to be 22% compared with 1% of the general population in the same study and 2.2% of the general population in other studies [37]. The 86-year-old man included had frequent outbreaks of eczematous dermatitis in the frontotemporal area of long evolution, with positive sensitization to benzyl salicylate present in the hairspray, sunscreen, and perfume he had used daily for years, and, by avoiding the allergen, his contact eczema resolved and his quality of life improved.

Other studies report a high incidence of sensitization and allergic contact dermatitis in patients with FFA, but the number of men included is very small [38,39].

## 4. Clinical

**Figure 2:**
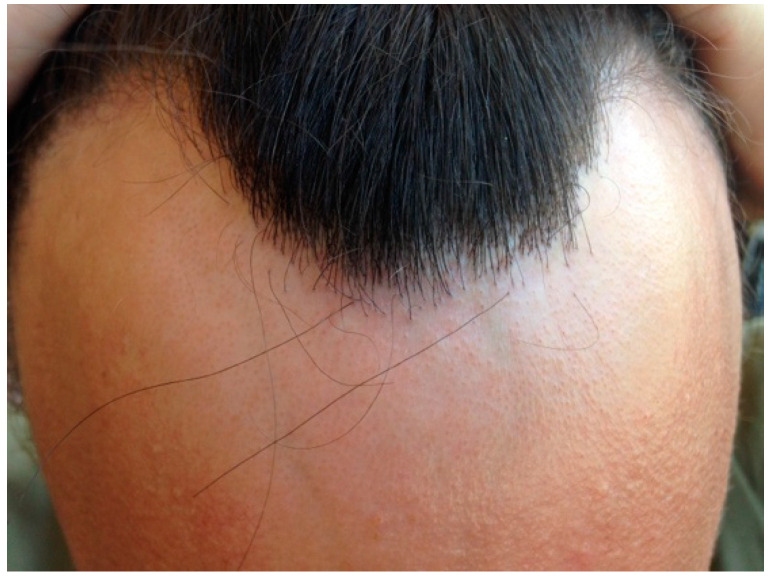
Recession of frontal hairline with lonely hairs and perifollicular erythema. The hairline shows a male receding hairline, compatible with previous or concomitant male androgenic alopecia.

Clinical signs in males with FFA are similar to those in females, with some peculiarities. The frequency of frontal hairline involvement is the highest (74% to 100%, in the longest series) (Figure 2), followed by temporal involvement (44% to 100%) [7,11,16,18].

**Figure 3:**
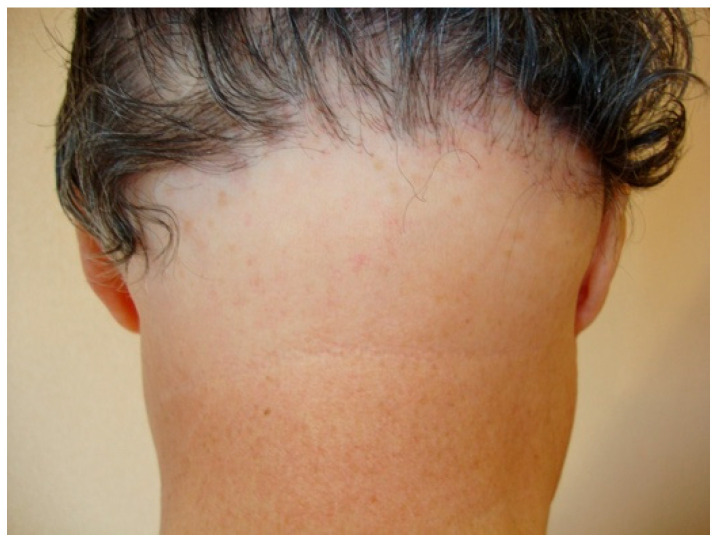
Occipital hair loss with perifollicular erythema.

Occipital involvement ranges from 8% in the series of Ormaechea et al. (n = 12) [11] to 45% in the series of Kanti et al. (n = 23) (Figure 3) [6]. Recession grades II and III have the highest prevalence in the series classifying the severity of FFA (Table 1) [8,11,17,40].

**Figure 4:**
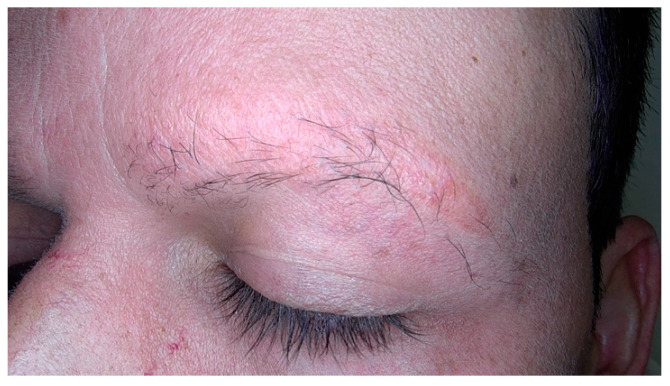
Partial loss of the eyebrows in a man with frontal fibrosing alopecia.

Regarding the loss of eyebrows, its prevalence varies in different publications, ranging from 43% [15] to 94.9% [13]. In general, most published case series have a very high percentage of eyebrow loss, which is very suggestive of male FFA (Figure 4). However, the prevalence of eyelashes loss in males with FFA appears to be low (2.6–8%) [7,13], even lower than in females (3–26%) [4,41].

**Figure 5:**
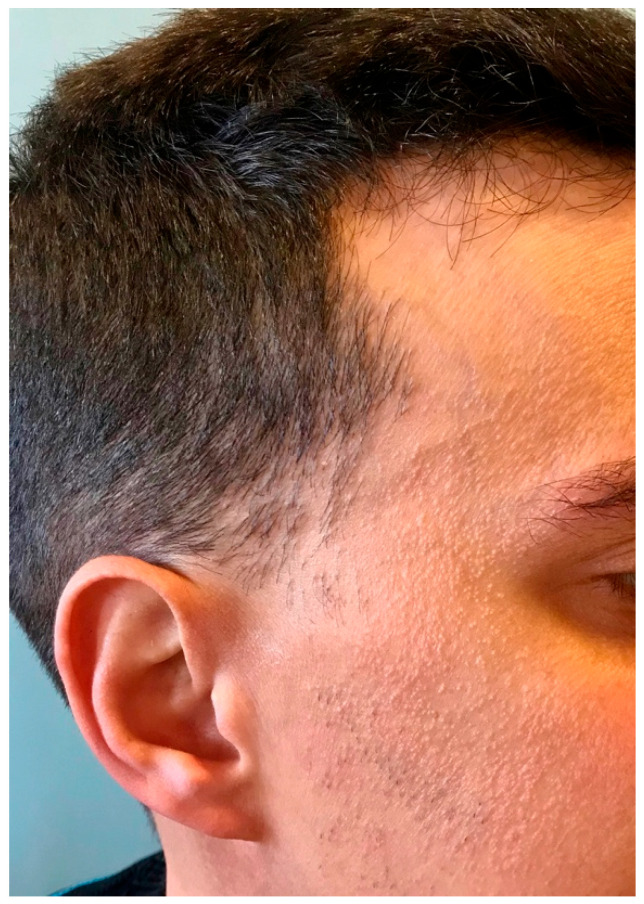
Man with frontal fibrosing alopecia, hair loss on sideburns and beard. Multiple facial papules can be observed on the cheeks and temples.

Facial papules appear to be a common finding in men with FFA (Figure 5). In the multicenter study by Vañó-Galván et al. [4], their presence is described in 33% of males versus 13.12% of females, similar to the prevalence described in the case series by Lobato-Berezo et al. (33.3%) [13]. In a retrospective study of 20 males with FFA published by Bernárdez C et al. [42], 70% of patients had facial papules, a higher frequency than was found in case series of females.

**Figure 6:**
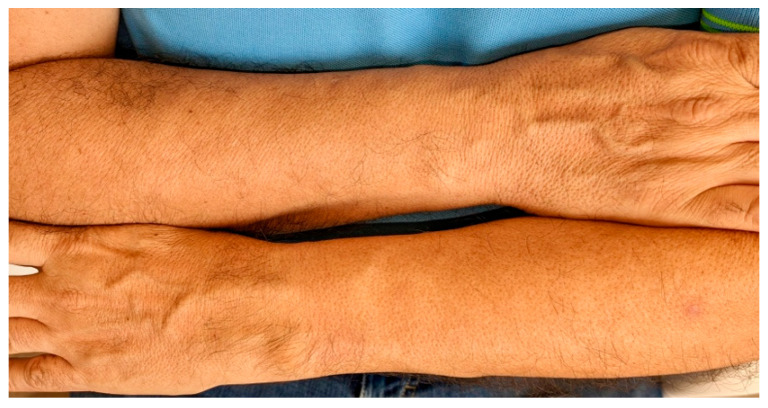
Loss of body vellus hairs on the upper limbs in a man with frontal fibrosing alopecia.

Body hair is affected in 29–83% of males (Figure 6) [4,7,10,12,14], higher than the 23.5% reported in females [4,41]. However, variable prevalences have been published for most of these signs, and the sample size of series of males with FFA is much smaller than that of females [4,16], so studies with more male patients would be desirable to confirm these data.

**Figure 7:**
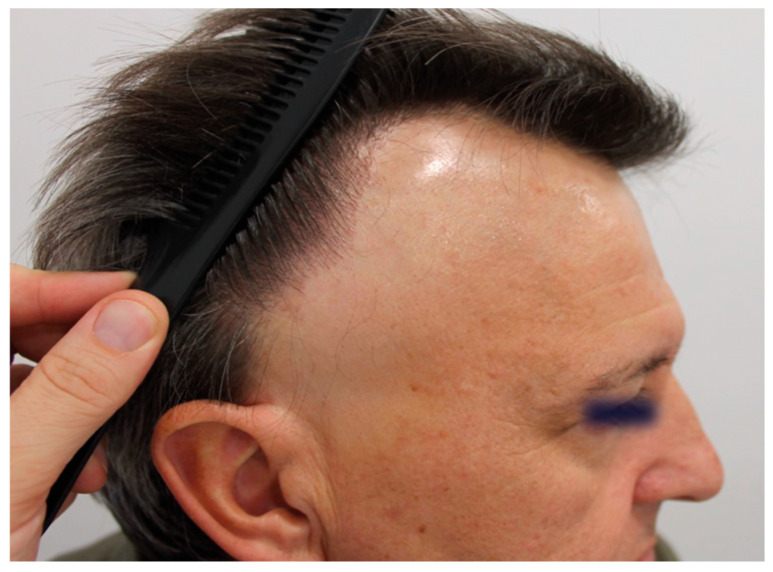
Frontal fibrosing alopecia pattern type I (linear). Frontotemporal recession of the hairline, hypopigmented cicatricial band and loss of the sideburns.

Among the clinical features commonly observed in men, early involvement of the beard and sideburns are common findings (Figure 7). However, prevalence data vary widely. Lobato et al. [13] describe beard and sideburn involvement in 74.4% and 89.7%, respectively, whereas, in the series of Pathoulas et al. [3], it is only 14.5% and 12.7%, respectively, the lowest percentages of all published series. This striking percentage difference may be due to the retrospective design of the study and the inclusion of patients with lichen planopilaris.

**Figure 8:**
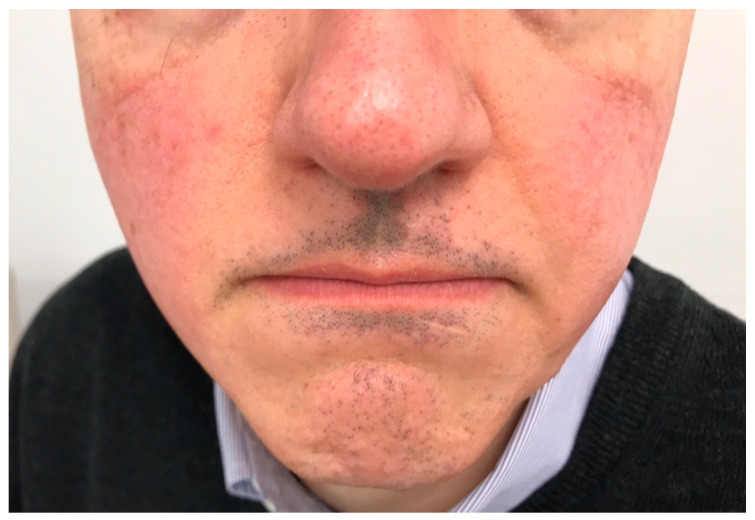
Loss of the beard on the cheeks, chin, and lateral mustache, with sparing of the central mustache.

Two clinical patterns of beard alopecia have been described: plaque alopecia, similar to alopecia areata, and diffuse involvement described by the patient as loss of density [12]. Bernárdez et al. [42] studied the clinical and trichoscopic features of beard alopecia in 20 men with FFA. The most commonly affected areas were the cheeks (100%), sideburns (90%), and moustache (90%), followed by the chin (60%). In patients with moustache involvement, 55.6% had predominantly lateral moustache involvement, sparing the central area (Figure 8).

**Figure 9:**
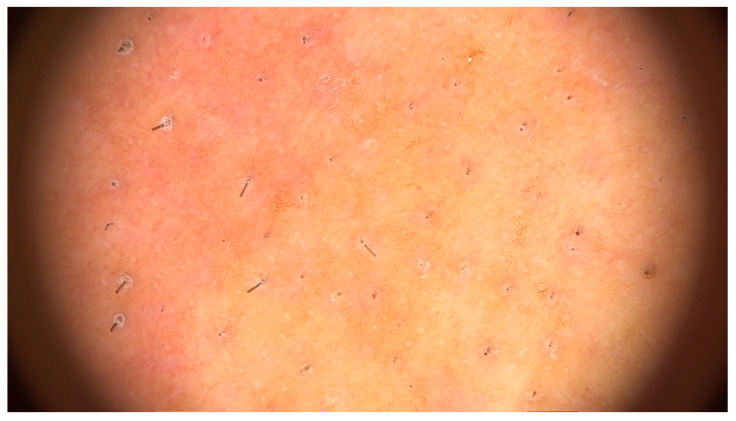
Trichoscopy of beard showing loss of follicular openings, erythema and perifollicular hyperkeratosis.

The most common trichoscopic signs in this anatomical region were the absence of follicular openings (100%), erythema (55.6%), and perifollicular hyperkeratosis (55.6%), followed by the presence of white dots (38.9%), black dots (23.1%), and yellow dots (16.7%) (Figure 9). Another common trichoscopic sign is the visualization of the proximal part of the hair shafts as they come to the surface through the transparency of the surrounding skin [61]. Trichoscopic features of the hairline are similar to those in women and include the loss of follicular openings (95–100%) [11,13], perifollicular erythema (62–71%) [3,6,13], and hyperkeratosis (34–74%) [6,13].

It should be noted that, although involvement of the beard and sideburns are common findings in men with the disease, these features are not part of the diagnostic criteria [62], but should be carefully analyzed when presenting a case of a man with alopecia in these facial areas, keeping in mind the possibility of FFA.

## 5. Diagnosis

**Figure 10:**
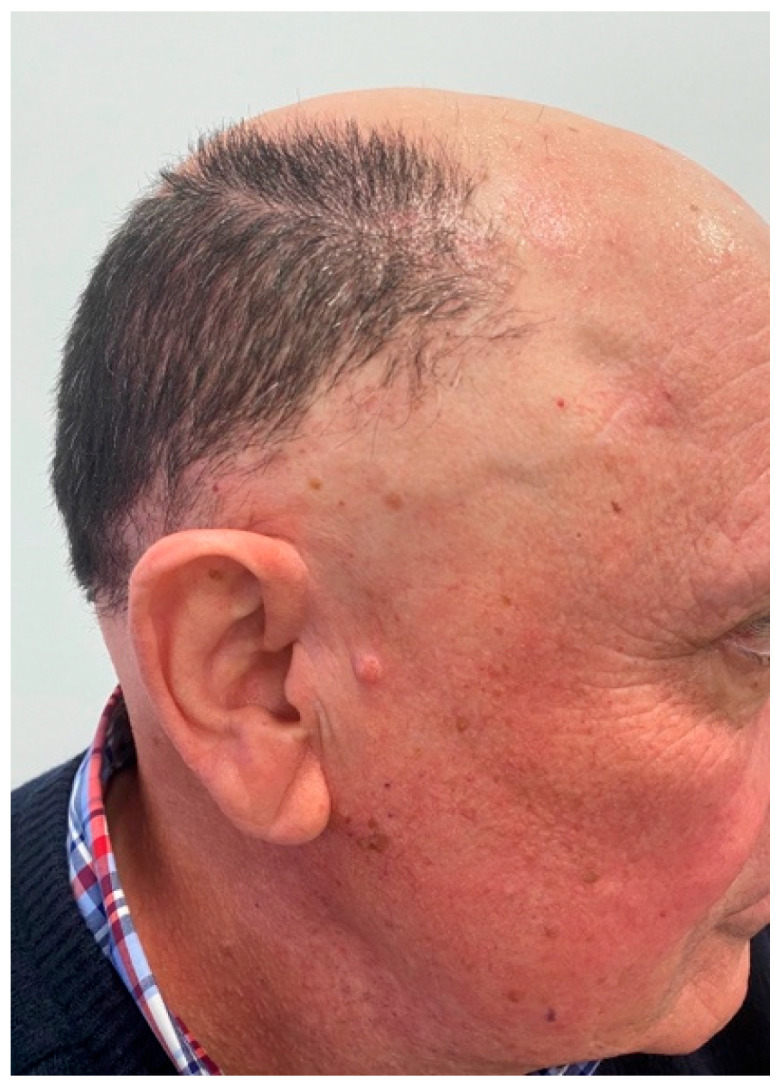
Man with advanced frontotemporal alopecia, loss of the eyebrows and beard. The hypopigmentation of the cicatricial band contrasts with the sun-exposed facial skin.

The diagnosis of FFA in males, as in females, is predominantly clinical. Bilateral partial or complete eyebrow alopecia, loss of sideburns, and beard alopecia are the three most important signs to suspect FFA due to their prevalence and characteristic presentation (Figure 10). The presence of facial papules or loss of body hair may also help to establish the diagnosis, and trichoscopy may be helpful in doubtful or incipient cases.

**Figure 11:**
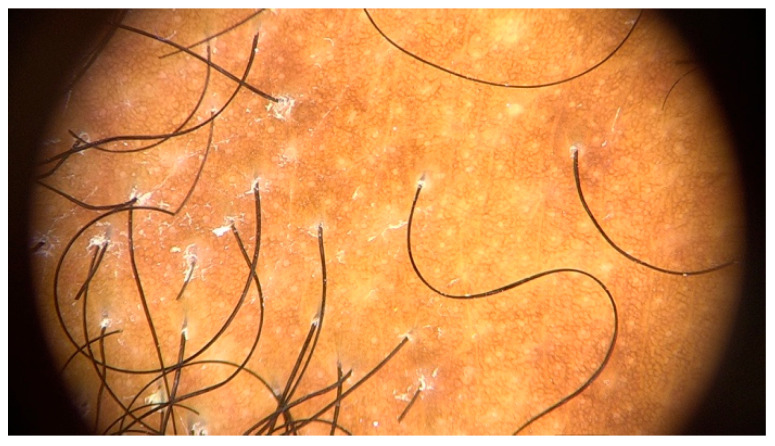
Trichoscopy on a sideburn of a black patient showing loss of follicular openings, loss of vellus hairs, and perifollicular hyperkeratosis.

Black patients have their own peculiarities, such as a lower prevalence described of FFA, an earlier age of onset, and often more severe clinical manifestations of the disease. Overlap with traction alopecia or central cicatricial centrifugal alopecia presents a diagnostic challenge. Trichoscopic peculiarities include less frequency of erythema or scaling, in addition to irregular distribution of white dots, and a honeycomb pigment network, different from what is usually seen on trichoscopy of FFA in caucasians (Figure 11) [63].

Biopsy may be required to confirm the diagnosis more often in men than in women, probably because of overlap with male AGA or differential diagnosis with AA (Table 2). Some case series have described high percentages of this test (25.6%) [13]. No studies have focused on identifying gender differences in the histopathological findings of FFA and they appear to be the same: the presence of a lymphocytic lichenoid infiltrate around the isthmus and infundibulum, including the bulge area, as well as concentric perifollicular lamellar fibrosis and atrophy of the sebaceous glands [64].

The International FFA Cooperative Group (IFFACG) [62] has developed consensus diagnostic criteria that assess frontotemporal, preauricular and scalp involvement, as well as the presence of inflammatory signs, facial papules and compatible biopsy (Table 3). However, these criteria have not yet been validated in men and do not include assessment of the beard (a sign of particular interest in this population).

A curious clinical sign was described in two cases of men with FFA. A diffuse vellus hair loss on the forearms, notably sparing the left wrist area constantly covered by their watch, termed the “watch sign”. Trichoscopic findings suggested FFA, and histopathology confirmed the diagnosis. The authors suggested a mechanism related to the Renbök phenomenon, in which the constant pressure exerted by the watch on the left wrist may influence the inflammatory process of the hair follicles, preserving them [43,65].

Another unusual report was that of spontaneous repigmentation of hair follicles (canities reversal) in a male patient with FFA [44], similar to what was described for seven women with FFA by Pastor-Nieto et al. [66]. The assumption is that the inflammatory infiltrate of the disease could have triggered hair repigmentation through the recruitment of residual melanocytes, a hypothesis that still needs to be proven.

## 6. Treatment

**Figure 12:**
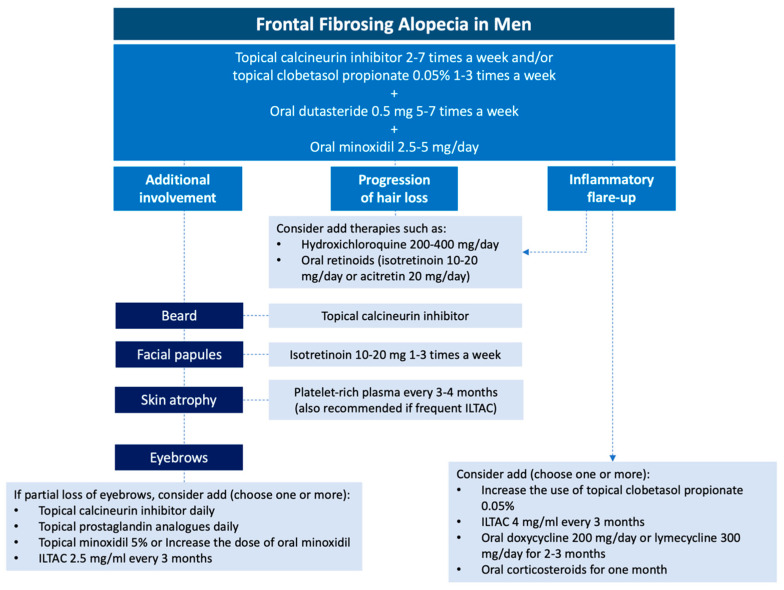
Therapeutic algorithm for male FFA proposed by the authors.

The therapeutic goal in FFA is to stabilize the condition and prevent further progression. Given the low prevalence of men with FFA reported in the literature to date, there are no randomized clinical trials evaluating the efficacy in this population. Currently, the therapeutic management of men is similar to that used in women, with oral 5-ARi, particularly dutasteride [29,67], combined with topical corticosteroids and topical calcineurin inhibitors as the first line of therapy (Table 4, Figure 12).

MAGA, male androgenetic alopecia; ILTAC, intralesional triamcinolone acetonide injections.

To date, the most commonly reported systemic treatment in men is hydroxychloroquine at doses of 200–400 mg/d, followed by 5-ARi (Table 5). The largest series on the use of these drugs in men is that of Moussa et al. [45], with a total of 12 patients: 7 on finasteride (mean daily dose 0.6 mg) and 5 on dutasteride (mean daily dose 0.4 mg). In general, the most commonly reported doses of finasteride range from 2.5 to 5 mg/day, while dutasteride doses range from 0.5 mg weekly or daily [69]. Pindado et al. [29] described better disease stabilization in the range of 0.5 mg dutasteride, 5 to 7 days per week.

In terms of topical therapy, corticosteroids are the most commonly used drugs in both sexes [4], specifically clobetasol propionate 0.05% [31,71], followed by betamethasone valerate or dipropionate [71]. The reporting of posology is uncommon in scientific articles, with a few exceptions. For instance, Peterson et al. [12] described the use of daily clobetasol propionate in six men, and Ormaechea et al. [11] described the application of betamethasone valerate in eight patients twice a week. The most commonly reported topical calcineurin inhibitor is tacrolimus in both women [31] and men (12 patients). The application schedule in men is only reported by Da Silva et al. [46] and refers to the use of pimecrolimus 1% twice a day in one patient.

The use of intralesional corticosteroids is reserved for areas with clinical or trichoscopic evidence of inflammatory activity. The most commonly used agent is triamcinolone acetonide every 3–6 months, at various concentrations ranging from 2.5–20 mg/mL [4,31,72]. The use of intralesional corticosteroids has been described in 16 men, in 11 of which triamcinolone acetonide is specified as the active ingredient at concentrations of 2.5 mg/mL (seven cases), 5 mg/mL every 6–8 weeks (one case) and 10 mg/mL (one case). Alegre-Sánchez et al. [8] reported a quarterly frequency of administration, but not the concentration. In addition, the use of oral tetracyclines can be considered to treat inflammatory flare-ups [12,45,73].

The coexistence of AGA, in up to 67% of cases, increases the frequency of adding topical or oral minoxidil to these treatment regimens [4,29,45,70]. Specifically, Moussa et al. [45] reported its use in 13 men with a dose range of 0.25–10 mg/d (mean 2 mg).

Low-dose isotretinoin is also commonly used to improve facial papules [74]. Doses range from 10 mg every other day to 20 mg daily [75]. The use of isotretinoin has only been reported in seven men, only two of whom reported a dose of 20 or 30 mg/d and clinical stability [12,47].

As with FFA in women, low-dose oral minoxidil [76], triamcinolone infiltration quarterly 2–10 mg/mL [72,77], and topical prostaglandin analogues such as bimatoprost 0.03% every 12 h may be used to treat eyebrow alopecia [78]. Zolkiewicz et al. [43] describe the use of tacrolimus and bimatoprost in the treatment of a man’s eyebrows, but not its outcome. Pham et al. [47] describe near-complete eyebrow regrowth in a man after 18 months of treatment with topical tacrolimus and isotretinoin 30 mg/d. On the other hand, AlGaadi et al. [64] report a patient with a single beard involvement who was treated with topical and intralesional corticosteroids (without specifying the active ingredient or dosage) and failed to achieve regrowth.

In recent publications, the safety of the use of platelet-rich plasma in scarring alopecia has been demonstrated. Its application can provide potential benefits as an anti-inflammatory treatment, slow the progression of alopecia, and improve cutaneous atrophy caused by the disease itself or by the use of corticosteroids [79,80].

From a surgical perspective, hair transplantation for patients with FFA is possible for those with stable disease who seek to enhance a small area. However, it is essential that patients are informed about the potential decline in graft survival over time. A retrospective study of 51 patients (3 men) with a mean stabilization period of 15 months showed a follicular unit survival rate of 41% at 5 years [81]. In the authors’ experience, the graft survival rate has improved in recent years, likely due to better therapeutic management of the disease before and after the surgery.

The first case of hair transplantation in a man with FFA was described by Kossard et al. [30] The patient who developed scarring alopecia 5 years after multiple interventions on the frontal hairline. Subsequently, Nusbaum et al. [48] published the results of surgery in a patient with a pre-existing diagnosis of FFA. After 10 months of clinical stability, a frontal graft was performed with a good response at 15 months. However, after 4 years, he had lost all grafts, even though he had stopped medical treatment. Hair transplantation in male FFA, as in women, should be considered after appropriate diagnosis and once the condition is stabilized under medical therapy. The patient’s expectations regarding the concept of improvement must be carefully managed, as FFA is a scarring condition that may have substantial psychosocial impact [82].

## 7. Conclusions

Frontal fibrosing alopecia (FFA) in men presents distinct clinical characteristics that often lead to misdiagnosis, particularly in cases of beard involvement. While the disease shares similarities with FFA in women, men are more likely to exhibit early beard and sideburn alopecia, facial papules, and body hair loss. The etiology remains unclear, but genetic, hormonal, and environmental factors, including the use of facial sunscreens and moisturizers, may have been implicated. Current diagnostic criteria, developed primarily for women, do not fully account for the unique presentations in men, highlighting the need for further research and validation. Treatment strategies largely mirror those used in female FFA, with 5-alpha reductase inhibitors, corticosteroids, and calcineurin inhibitors as first-line options. However, more studies are required to optimize treatment protocols and improve outcomes in this underrecognized population.

## Figures and Tables

**Table 1:** Clinical characteristics of men with FFA reported in the literature.

Clinical Feature	N (%)
Affected area	
• Frontal (n = 114) ^a^	108 (94.7)
• Temporal (n = 114) ^a^	100 (87.7)
• Occipital (n = 188) ^b^	39 (20.7)
Degree of recession (n = 36) ^c^	
• I (<1 cm)	3 (8.3)
• II (1–2.99 cm)	14 (38.9)
• III (3–4.99 cm)	8 (22.2)
• IV (5–6.99 cm)	3 (8.3)
• V (≥7 cm)	3 (8.3)
Inflammatory signs (n = 208) ^d^	
• Perifollicular erythema	139 (66.8)
• Perifollicular hyperkeratosis	118 (56.7)

^a,b^ N calculated from 31 articles specifying frontotemporal and occipital involvement [2,4,6,7,11,12,13,15,16,18,26,28,30,31,32,36,41,42,43,44,45,46,47,48,49,50,51,52,53,54,55,56,57,58]. ^c^ N calculated from 7 articles reporting the degree of recession [4,11,17,36,40,46,48,55]. ^d^ N calculated from 25 articles reporting the presence of inflammatory signs [2,3,6,11,12,13,15,16,17,26,28,36,40,45,46,48,49,50,52,53,54,55,56,59,60].

**Table 2:** Differential diagnosis of male frontal fibrosing alopecia.

Clinical Feature	FFA	AA	MAGA
Trichoscopy	Loss of follicular openings, perifollicular erythema, perifollicular hyperkeratosis	Black dots, yellow dots, exclamation marks hairs	Anisotrichosis, preserved vellus hair
Patchy alopecia	Possible (concomitant LPP)	Frequent	Absent
Beard alopecia	Frequent (patchy or diffuse)	Possible (patchy)	Absent

FFA, frontal fibrosing alopecia; AA, alopecia areata; MAGA, male androgenetic alopecia; LPP, lichen planopilaris.

**Table 3:** Diagnostic criteria of the International FFA Cooperative Group (IFFACG) [62].

CLASSIC FFA ^a^	PROBABLE FFA ^a^
Frontal recession with loss of follicular openings (2)	Frontal recession without loss of follicular openings (1)
Biopsy consistent with FFA in frontotemporal region or eyebrows (2)	
Eyebrow loss ≥ 50% (in absence of alopecia areata) (1)	
Perifollicular anterior scalp erythema (1)	
Perifollicular anterior scalp hyperkeratosis (1)	
	Facial papules (1)
	Bilateral preauricular hair loss (1)
	Absence of vellus hair on frontotemporal line (1)

^a^ Must score at least 4 points to meet criteria for classic or probable FFA. These diagnostic criteria have not been validated in men.

**Table 4:** Treatments administered to men with frontal fibrosing alopecia reported in the literature.

Study	N	Treatment	N (%)	Combination of Treatments	Response Data
Kanti et al., 2019 [6]	23	Topical CS	6 (29)	NS	NS
		ILCS	3 (16)		
		Tetracyclines	3 (16)		
		Finasteride	2 (11)		
		Systemic CS	1 (5)		
		HXQ	1 (5)		
		MTX	1 (5)		
		Oral clindamycin	1 (5)		
Rayinda et al., 2022 [16]	17	Topical CS	8 (47)	NS	NS
		HXQ	8 (47)		
		TCI	6 (35)		
		Isotretinoin	4 (24)		
		Systemic CS	1 (6)		
Moussa et al., 2022 [45]	13	Oral minoxidil	13 (100)	Minoxidil + topical CS	1/1 (8 months)
		Finasteride	7 (54)	Finasteride + minoxidil	3/4 stable (7 months)
		Dutasteride	5 (39)	Dutasteride + minoxidil	1/2 stable (3 months)
		Topical CS	4 (31)	Dutasteride + minoxidil + topical CS	1/2 stable (4 months)
				Finasteride + minoxidil + topical CS	0/1
		TCI	1 (8)	Dutasteride + minoxidil + topical CS + TCI	0/1
		Tetracyclines	1 (8)	Finasteride + minoxidil + minocycline	1/1 (9 months)
		CsA	1 (8)	Finasteride + minoxidil + CsA	1/1 (3 months)
					8/13 stable in 6 months
Alegre-Sánchez et al., 2017 [8]	12	Topical CS	8 (67)	Topical CS + topical minoxidil	Improvement 2/6, stable 2/6, progression 2/6
		Topical minoxidil	8 (67)		
		HXQ	2 (17)	HXQ 200 mg/d	Stable (15 y 17 months)
		ILCS	1 (8)	Quarterly ILCS + topical CS + topical minoxidil	Stable (12 months)
		Systemic CS	1 (8)	Prednisone 0.5 mg/kg/d	Stable
		Finasteride	1 (8)	Finasteride 1 mg + topical CS+ topical minoxidil	Stable
Ormaechea et al., 2016 [11]	12	Topical CS	8 (66)	NS	Stable (20 months)
		Topical minoxidil	4 (33)		
Peterson et al., 2020 [12]	7	Topical CS	7 (100)	Topical CS + ILCS + minoxidil	Stable (4 months)
		ILCS	7 (100)	Topical CS + ILCS + minoxidil + TCI + doxycycline	Stable (1 month)
		TCI	5 (71)	Topical CS + ILCS + TCI + doxycycline	Stable (14 months)
		Topical minoxidil	5 (71)	Topical CS + ILCS + minoxidil + TCI + finasteride	Stable (4 months)
		Finasteride	3 (43)	Topical CS + ILCS + minoxidil + finasteride	Stable (10 months)
		Tetracyclines	4 (57)	Topical CS + minoxidil + doxycycline	Progression *(change to next treatment)*
		Pioglitazone	2 (29)	Topical CS + ILCS + minoxidil + TCI + Finasteride + HXQ + pioglitazone + naltrexone + PRP	Stable (51 months)
		Naltrexone	1 (14)		
		PRP	1 (14)		
		HXQ	1 (14)		
		Isotretinoin	1 (14)	Topical CS + ILCS + minoxidil + TCI + pioglitazone + isotretinoin	Stable (17 months)
Tolkachjov et al., 2017 [15]	7	Topical CS	6 (86)	Topical CS	Monotherapy: 3 progression, 1 stable (3 weeks)
		HXQ	4 (57)	HXQ + TCI + topical CS	1 progression (4 months), 1 stable (2 months)
				HXQ + TCI	1 stable (4 months)
				HXQ + topical CS	1 stable (6 months)
		TCI	3 (43)	TCI	Monotherapy: 1 progression
		MMF	1 (14)	MMF	Monotherapy: stable (3 months)
		ILCS	1 (14)	ILCS + topical CS	Progression
Dlova et al., 2015 [68]	1	Topical CS + TCI + topical minoxidil + HXQ			Stable (12 months)
Nusbaum et al., 2010 [48]	1	Topical CS + ILCS + finasteride + HXQ			Stable (10 months)
Chen et al., 2014 [50]	1	Topical CS + systemic CS + topical minoxidil			Stable (3 months)
Pham et al., 2020 [47]	1	Topical CS + ILCS + HXQ			Progression
		ILCS + TCI + isotretinoin			Eyebrows regrowth (18 months)
Salido-Vallejo et al., 2014 [52]	1	Topical CS + systemic CS + topical minoxidil + HXQ + acitretin			Progression
Roche et al., 2008 [28]	1	Topical CS			Progression
AlGaadi et al., 2015 [60]	1	Topical CS + ILCS			Only beard affected, no regrowth
Da Silva et al., 2018 [46]	1	Topical CS + TCI			Progression (7 years)
White et al., 2016 [53]	1	Topical CS			NS
Banka et al., 2014 [31]	1	Finasteride			NS
Singh et al., 2022 [55]	1	TCI + Finasteride + HXQ			NS
Barreto et al., 2022 [44]	1	Topical CS + ILCS + topical minoxidil			NS
Starace et al., 2022 [56]	1	Systemic CS + topical minoxidil + TCI			NS
Zolkiewicz et al., 2024 [43]	1	Topical CS + ILCS + TCI + oral minoxidil + isotretinoin + bimatoprost			NS

CS, corticosteroids; ILCS, intralesional corticosteroids; HXQ, hydroxycloroquine; MTX, methotrexate; TCI, topical calcineurin inhibitors; CsA, cyclosporine; PRP, platelet-rich plasma; MMF, mycophenolate mofetil; NS, not specified.

**Table 5:** Summary of treatments, dosage, and outcomes for male AFF reported in the literature.

Treatment	N	Active Ingredient and Dosage	Stop Progression
Topical CS	57	Clobetasol propionate 0.05% (19) Hydrocortisone butyrate (1) [12] Betamethasone valerate twice weekly (8) [11] Halcinonide 0.1% BID (1) [48] NS (28)	29/38 (NS 19)
Oral minoxidil	24	0.25–10 mg/d (median 2 mg) (13) [45] NS (11) [43,70] 07/03/2025 10:32:00	8/13 (NS 11)
Topical minoxidil	24	2% (1) [68] 5% (14) ○ BID (7) [12,46] ○ Daily (4) [11] ○ NS (3) NS (9)	14/18 (NS 6)
TCI	22	Tacrolimus (12) ○ 0.1% (4) [12,47,56,68] ○ 0.3% (4) [12] ○ NS (4) Pimecrolimus (4) ○ 1% BID (1) [46] ○ NS (3) NS (6)	9/12 (NS 10)
HXQ	20	200 mg/d (3) [8,12] 200 mg/12h (3) [48,55,68] NS (14)	8/10 (NS 10)
ILCS	16	Triamcinolone (11) ○ 2.5 mg/mL (7) [12,48] ○ 5 mg/mL every 6–8 weeks (1) [47] ○ 10 mg/mL (1) [12] ○ NS (2) NS (5)	10/11 (NS 5)
Finasteride	15	1 mg/d (6) [12,48] 0.25–1 mg/d (median 0.6 mg) (7) [45] NS (2)	10/11 (NS 4)
Tetracyclines	8	Doxycycline (4) [12] ○ 100 mg/d (3) ○ 200 mg/d (1) Minocycline (1) [45] NS (3)	4/5 (NS 3)
Systemic retinoids	8	Isotretinoin (7) ○ 20 mg/d (1) [12] ○ 30 mg/d (1) [47] ○ NS (5) Acitretin (1) [52] 07/03/2025 10:32:00	2/3 (NS 5)
Systemic CS	6	IMCS 40 mg/month 3 months (1) [56] Prednisolone 1 mg/kg 2 weeks (1) [50] Prednisone 0.5 mg/kg/d (1) [8] NS (3)	2/3 (NS 3)
Dutasteride	5	0.25–0.5 mg/d (median 0.4 mg) (5) [45]	2/5
Pioglitazone	2	30 and 15 mg/d [12]	2/2
Naltrexone [12]	1	4.5 mg/d	1/1
CsA [45]	1	NS	1/1
MMF [15]	1	NS	1/1
MTX [6]	1	NS	NS
Oral clindamycin [6]	1	NS	NS

CS, corticosteroids; TCI, topical calcineurin inhibitors; HXQ, hydroxycloroquine; ILCS, intralesional corticosteroids; CsA, cyclosporine; MMF, mycophenolate mofetil; MTX, methotrexate; IMCS, intramuscular corticosteroids; NS, not specified; BID, twice a day. * Total of male patients: 115.

## Data Availability

Data sharing is not applicable to this article.
